# Seamless and intuitive control of a powered prosthetic leg using deep neural network for transfemoral amputees

**DOI:** 10.1017/wtc.2022.19

**Published:** 2022-09-28

**Authors:** Minjae Kim, Ann M. Simon, Levi J. Hargrove

**Affiliations:** 1 Department of Physical Medicine and Rehabilitation, Northwestern University, Evanston, IL, USA; 2 Center for Bionic Medicine, Shirley Ryan AbilityLab, Chicago, IL, USA

**Keywords:** ambulation modes, deep learning, prosthetics, impedance control, an open-source bionic leg

## Abstract

Powered prosthetic legs are becoming a promising option for amputee patients. However, developing safe, robust, and intuitive control strategies for powered legs remains one of the greatest challenges. Although a variety of control strategies have been proposed, creating and fine-tuning the system parameters is time-intensive and complicated when more activities need to be restored. In this study, we developed a deep neural network (DNN) model that facilitates seamless and intuitive gait generation and transitions across five ambulation modes: level-ground walking, ascending/descending ramps, and ascending/descending stairs. The combination of latent and time sequence features generated the desired impedance parameters within the ambulation modes and allowed seamless transitions between ambulation modes. The model was applied to the open-source bionic leg and tested on unilateral transfemoral users. It achieved the overall coefficient of determination of 0.72 with the state machine-based impedance parameters in the offline testing session. In addition, users were able to perform in-laboratory ambulation modes with an overall success rate of 96% during the online testing session. The results indicate that the DNN model is a promising candidate for subject-independent and tuning-free prosthetic leg control for transfemoral amputees.

## 1. Introduction

One of the greatest challenges to achieving a meaningful impact on the lives of amputees is the development of safe, robust, and intuitive control strategies for robotic prostheses. Amputation of the lower limb, affecting an estimated 1 million Americans (Ziegler-Graham et al., 2008) causes profound disability – significantly limiting mobility, independence, and potentially the ability to pursue employment or leisure activities. A new category of powered joints, including powered knees and ankles, may allow for better outcomes, such as enabling users to walk across more challenging terrains (e.g., walking up and down hills, ramps, and stairs) more efficiently than using passive prostheses (Montgomery and Grabowski, 2018; Kim et al., 2021). However, to take advantage of the device capabilities, it is critical that they are controlled properly.

Modern self-contained powered-leg devices are generally constructed from strong, lightweight materials and use a battery-powered brushless-DC motor(s) (Au et al., 2008; Lawson et al., 2014). These devices generally include multiple sensors within their construction to provide information with regard to the current state of the devices. To provide actuation, the current is commanded to the motor, and the current is then transformed into a torque produced at the shaft of a motor. The motor torque is further amplified by the transmission of the device to result in torque available at the appropriate joint. Commercially available brushless-DC motor controllers can be purchased to ensure that the commanded current is properly executed by the motor (using hall effect sensors and ensuring that the motor is commutated properly). Well-established control principles from the robotics field can be used to allow for the calculation of the motor current required to achieve a certain position, velocity, force, torque, or impedances (e.g., allowing the system to behave like a virtual spring damper system). These are all examples of *low-level controllers.* Choosing the desired value that needs to be controlled (e.g., position trajectory, impedance values, and so forth) remains a more challenging problem in controlling assistive devices.

Determining what commands to send to the low-level controller is usually achieved using a finite-state machine. It is relatively straightforward to create a small and self-contained finite-state machine to implement walking (Sup et al., 2008). This is often termed an *intrinsic* or *mid-level controller.* For example, a load cell can easily be used to separate the stance and swing phases of gait, and joint velocity can be used to differentiate between swing flexion and swing extension. Next, each joint can be modeled as a set of virtual impedance parameters in every sub-phase. When all of the parameters are carefully tuned, usually by hand (Simon et al., 2014), but more recently with machine learning approaches (Huang et al., 2016) or deep learning techniques (Wen et al., 2019), a smooth gait can be generated by the user. Alternative approaches can be used to generate mid-level controllers that are less reliant on finite-state machines. For example, prosthesis control strategies that mimic the underlying dynamics and control policies of the human neuromuscular system can be created (Thatte and Geyer, 2015). Instead of approximating the behavior of individual human leg joints with impedance or similar functions, they model the human control system that generates this behavior (Song and Geyer, 2015). Another alternative is to use a phase-based mid-level control architecture (Quintero et al., 2018). Using this approach, the gait cycle is typically viewed as a periodic sequence of events over time, starting with heel contact during the initial stance and ending with knee extension during a late swing. Recent work has shown that a mechanical variable, specifically the phase angle of the hip joint, can accurately parameterize human leg patterns across perturbations and be used to control a powered leg during walking (Villarreal and Gregg, 2016; Villarreal et al., 2016).

Regardless of the style of mid-level control, the difficulty in creating a finite-state machine is compounded when more activities need to be restored. This is especially true when allowing seamless and automatic transitions between activities. This is usually achieved using a *high-level controller.* Examples of high-level controllers include key-fobs, threshold-based rules (Xu et al., 2018), or machine learning algorithms (Liu et al., 2017) that automatically predict which low-level controller should be executed. In addition, a human-inspired variable-based prediction method (Quintero et al., 2017) was proposed.

The user interacts directly with their device (and indirectly with the environment) through the socket. The device responds to these interactions through the control system using the previously described approach. Rather than imposing specific mid- or high-level controllers, we believe that an alternative approach to solving this problem would be to use deep-learning tools to create a direct mapping between the sensors available on the device and the motor commands required to restore locomotion. Machine learning, including statistical pattern recognition classifiers and artificial neural networks, has been used to help control upper-limb prostheses for decades (Graupe et al., 1982; Hudgins et al., 1993) and has recently been applied to infer user intention as part of the high-level controller for powered leg prostheses (Huang et al., 2011; Young et al., 2013). Ambulation modes were estimated by analyzing muscle activation patterns from ultrasound images (Rabe et al., 2020) or electromyography signals (Spanias et al., 2018).

Deep neural networks (DNNs) include several layers that can be connected in a variety of ways depending on the applications. DNNs enable end-to-end learning from inputs to outputs with little or no prior knowledge, and this simplifies the system. The input data space, which is usually of high dimension, can be encoded into a lower-dimensional hidden (or latent) space to uncover structure within the data. The latent space can then be classified to determine user intention or, more relevantly for this application, decoded into a set of continuous outputs. DNNs have recently been demonstrated in a wide range of applications, including several in the powered prosthetics and orthotics field. For robust, high-level control decisions, convolutional neural networks (CNNs) were used for vision-based environment sensing to predict ambulation modes prior to physical movement (Laschowski et al., 2022). A CNN-based method (Su et al., 2019) using inertial measurement units (IMUs) was used to predict 13 ambulation modes, including transitional states. A stacked autoencoder was employed to estimate multi-degrees of freedom wrist torque (Yu et al., 2020) based on EMG signals, and it outperformed the conventional regression methods for eight able-bodied subjects and one amputee subject. A multi-stream long short-term memory (LSTM) dueling model (Ren et al., 2019) has been proposed to predict arm trajectory based on EMG signals and IMU data for control of an upper limb rehabilitation exoskeleton. For control of a robotic hip exoskeleton (Kang et al., 2021), CNNs and LSTM networks were used to estimate the human gait phase and generate desired hip torque for various ambulation modes: level-ground walking (LGW), ascending/descending stairs (SA/SD), and ascending/descending a ramp (RA/RD). Although this approach showed accurate performance of about 5% phase estimation error between actual and normative trajectories for 10 subjects, the model was trained on a user-specific basis (i.e., all 10 individual models for 10 subjects). Our group has used DNNs with data augmentation to learn robust representations of movement intention for powered leg prostheses (Hu et al., 2019) and to improve classification performance in the presence of noise for EMG-based control of upper-limb prosthetic limbs (Teh and Hargrove, 2021).

In this article, we propose a DNN-based unified model to control prosthetic legs for transfemoral amputees. We demonstrated that the DNN model (i) extracts latent features to distinguish ambulation modes and time sequence features to recognize the gait phase; (ii) facilitates control for five ambulation modes with seamless transition between modes; (iii) facilitates subject-independent and tuning-free control.

## 2. Methods

### 2.1. Powered prosthetic leg

Data collection and online evaluation of the DNNs were performed using a self-contained open-source bionic leg (OSL) (Azocar et al., 2020). The OSL includes a powered knee and ankle joint (actuation along the sagittal plane only) and has a mass of 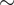 4,000 g. Actuation at each joint is provided using exterior rotor brushless DC motors with a closed loop controller (Dephy Inc., Maynard, MA) powered by a 36 V lithium-polymer battery back. The knee uses a 3-stage belt-drive transmission, and the ankle uses a 2-stage belt-drive transmission in series with a single-stage kinematic linkage. The OSL also includes several sensors which can be used to monitor the state of the device. For this experiment, we collected data from 18 mechanical sensors (Table 1) embedded in the OSL: data of the six-axis force/moment, knee/ankle joint angles, and velocities, acceleration and angular velocity, and thigh/shank angles.

**Table 1.** Configuration of mechanical sensors

Signals	Sensor description	Sampling frequency (Hz)
Force (*N*) and moment (Nm)	six-axis load cell	500
Knee/ankle angles (deg) and velocities (deg/s)	Joint encoder	250
Accelerations (*g*) and angular velocities (deg/s)	IMU mounted on the knee	250
Thigh/shank angles (deg)	Mathematically calculated inclinations by using joint angles and IMU signals	250

Abbreviation: IMU, inertial measurement unit.

We implemented an impedance controller such that the motors generated torque for the knee and ankle based on the following equation:(1)

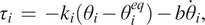

where 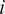 represents the knee or ankle joint, 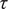 represents the joint torque, 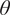 and 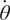 represent the joint angle and velocity, respectively, and 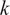 , 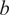 , and 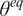 denote the stiffness, damping coefficient, and equilibrium angle, respectively. The knee joint measured zero at full extension, and knee flexion was measured as a positive value. Sign convention for ankle dorsiflexion was positive, and ankle plantar flexion was negative. In the case of the thigh, extension was positive, and flexion was negative. Additional details with respect to the hardware, available sensors, and impedance controller can be found in Azocar et al. (2020).

### 2.2. State machine

A state machine-based impedance controller (Simon et al., 2014) was used to collect training data. The state machine was configured to include five ambulation modes: LGW, SA/SD, and RA/RD. Generally, each ambulation mode was subdivided into four states corresponding to early stance, late stance, early swing, and late swing. The impedance parameters within each state were adjusted in each session based on a combination of user feedback and visual inspections of the kinematics conducted by the prosthetist and therapist. The latest range of impedance parameters by ambulation mode and phase for all users can be found in the Supplementary Material.

State transitions between individual states were configured using the mechanical sensors on the limb to allow for seamless transitions between activities. The state machine ran on an embedded controller and updated impedance parameters every 25 ms. Additional details on the finite-state machine and configuration process can be found in Simon et al. (2014 and 2016).

### 2.3. Deep neural network

**Figure 1.**
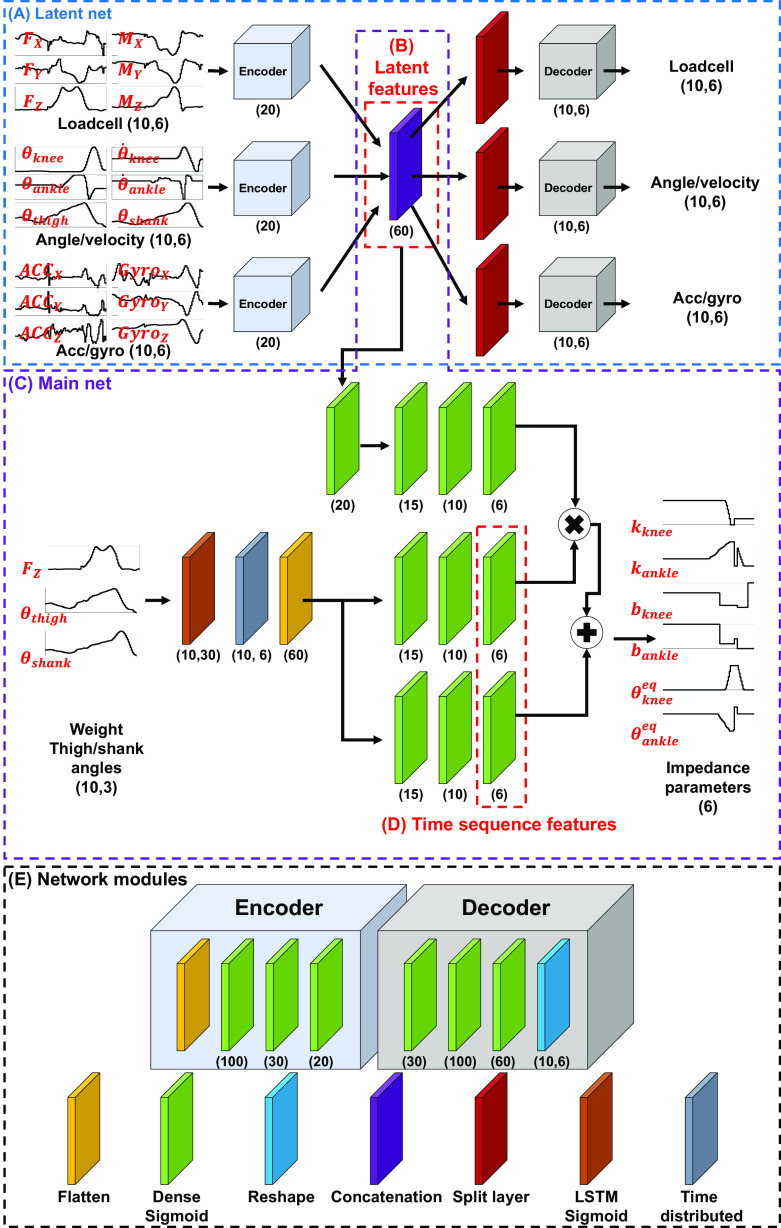
Architecture of the proposed DNN. The model takes a history of 18 mechanical sensor datapoints (see Table 1) as the inputs to obtain the single-time step impedance parameters. The latent network (a) extracts latent features (b). The latent features and history of three sensor data (weight, thigh angle, and shank angle) are used as the inputs of the main network (c) to extract time sequence features (d) and output impedance parameters. (e) The network modules represent the function of layers. The number in the brackets represents the number of units in the layers (e.g., Dense (*N*) represents the Dense layer with *N* units, and Load cell (*Hist*, *N*) represents *N* load cell sensor data points of the *Hist*-time step).

The overview of the proposed DNN architecture is presented in Figure 1. Network modules include flattening, dense sigmoid, reshaping, concatenation, split, LSTM sigmoid, and time distributed modules (Figure 1e). An encoder module and decoder module were constructed from these modules. The encoder module maps the input data to lower-dimensional latent features, and the decoder module maps the encoded latent features back to the original input data. These modules were arranged to create a DNN comprised of two sub-networks: the latent and main networks; the networks have a total of 37,601 trainable parameters. The hyperparameters in the networks, such as units of the dense layers, were heuristically chosen.

1. Latent network: The purpose of the latent network is to extract latent features that discriminate the differences between the ambulation modes. The latent network (Figure 1a) extracts latent features (Figure 1b) from 50 ms of data recorded from all 18 sensors, using the encoder and decoder. The network was trained for 50 epochs (batch size: 256, RMSprop (Hinton et al., 2012) with a learning rate of 0.001 and the loss function of the mean squared error (MSE)). At each epoch, the data were shuffled and split into training and validation data with a ratio of 7:3.
2. Main network: After the encoder parameters were identified, the main network (Figure 1c), including the LSTM network, was trained. The purpose of the LSTM network is to extract discriminant latent features that change sensitively during the gait cycle. The LSTM network only used the user weight, thigh angle, and shank angle among the 18 sensors. After that, the time sequence features (Figure 1d) and the latent features (Figure 1b) were combined to extract output impedance parameters. This main network was trained with the following learning configuration: 50 epochs, batch size: 256, RMSprop with a learning rate of 0.001 and the loss function of the MSE). At each epoch, the data were shuffled and split into training and validation data with a ratio of 7:3.

Here, all sensor data and impedance parameters were scaled to values between 0 to 1 for application to the DNN model. In the case of the sensor data, forces and moments were normalized by weight; joint angles and velocities were divided by 36 and 500, respectively; and accelerations and angular velocities were divided by 0.8 and 200, respectively. Then, additional scaling was applied as follows:(2)

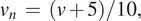

where 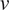 denotes the raw sensor data, and 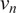 denotes the normalized data that is bounded from 0 to 1 centered at 0.5.

Additionally, the output impedance parameters from the DNN models were limited (Table 2) for safety.

**Table 2.** The upper and lower limits of the impedance parameters

	Knee parameters			Ankle parameters		
	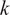 (Nm/deg)	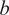 (Nm  s/deg)	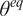 (deg)	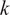 (Nm/deg)	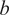 (Nm  s/deg)	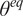 (deg)
Upper limit	12	0.6	110	15	0.6	16
Lower limit	0	0	0	0	0	−12

**Figure 2.**
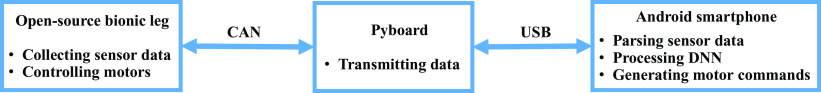
System configuration. The Pyboard was connected with the OSL and the Android smartphone, respectively, by CAN and USB. The Pyboard acted as a bidirectional translation module between the smartphone and the OSL.

The pairs of sensor data (i.e., network inputs) and impedance parameters (i.e., network outputs) calculated by the state machine were used to train the DNN model with TensorFlow (v. 2.4.1, Google) in Python on a laptop (ROG Strix G17, ASUS) under Windows 10 with NVIDIA GeForce RTX 2070 SUPER GPU. The trained DNN model was deployed to an Android 10 smartphone (Galaxy Note 9, Samsung) and ran on a CPU (Exynos 9810). The execution time of the proposed DNN on the smartphone was about 1.2 ms, and it is sufficient to control prosthetic legs in real time. A small microcontroller module (Pyboard D-Series) acted as a bidirectional translation module between the smartphone and the OSL. Its purpose was simply to accept commands from the phone over USB serial communication and transmit them to the OSL via CAN and vice versa. The smartphone parsed sensor data and generated impedance parameters every 5 ms, but the parameters could be transmitted to the leg only every 25 ms due to limitations in the translation board. The proposed system diagram is shown in Figure 2.

### 2.4. Experimental protocol

Six individuals (TF1–6) with unilateral transfemoral amputation participated in this study (Table 3). All individuals provided written informed consent to a protocol approved by the Northwestern University Institutional Review Board. TF1 and TF6, who had less experience (<4 hr) walking on the OSL, were considered novice users; and TF2–5, who had experience (>10 hr) walking on the OSL, were considered experienced users. All users were fit with the prosthesis by a certified prosthetist and instructed by a licensed therapist. The users performed in-laboratory ambulation modes, including LGW at self-selected speed, SA and SD on a six-step staircase, and RA and RD on a 10° inclined surface.

**Table 3.** Subject demographics

User	Gender	Age (years)	Time post-amputation (years)	Etiology	Height (m)	Weight (kg)	Previous OSL experience (hr)
TF1	M	36	24	Left sarcoma	1.87	86.2	approx. 4
TF2	F	54	32	Right trauma	1.65	71.0	>20
TF3	M	31	8	Right sarcoma	1.93	72.6	>20
TF4	F	34	20	Right sarcoma	1.7	70.3	>10
TF5	M	36	17	Left sarcoma	1.93	104	>10
TF6	F	35	29	Left sarcoma	1.7	72.5	approx. 3

Abbreviation: OSL, open-source bionic leg.

The collected datasets consisted of three cases: training data, offline testing data, and online testing data. The training data and offline testing data were obtained with the state machine control; they were collected separately on different days (i.e., the offline testing data were not included in the training data). During these sessions, we changed the ambulation mode manually using a key fob to prevent incorrect transitions between different ambulation modes. The online testing data were obtained with the DNN model. During each session, we collected as much data as possible as experimental time allowed per subject. In total, 5,417, 1,127, and 744 steps on all ambulation modes were collected for training, offline testing, and online testing, respectively. The detailed number of steps per user is shown in Table 4.

**Table 4.** Collected datasets for training, offline testing, and online testing

	The number of steps (LGW/SA/SD/RA/RD)		
User	Training	Offline testing	Online testing
TF1	252/11/18/20/33	—	—
TF2	1,524/55/81/129/151	—	93/23/6/90/101
TF3	1,490/75/130/157/209	307/36/75/56/66	102/15/21/25/27
TF4	789/60/91/62/80	—	—
TF5	—	166/13/17/33/42	173/18/16/13/21
TF6	—	206/17/26/21/46	—

Abbreviations: LGW, level-ground walking; RA/RD, ascending/descending a ramp; SA/SD, ascending/descending stairs.

A single unified DNN model was trained using all ambulation mode data from all training users. Model training took about 312 min. Subsequently, the DNN model was applied to the offline testing data to compare output parameters to those of the state machine.

In the online testing session, the single unified DNN model controlled the OSL for all online testing users. During online testing, at least five repetitions of each ambulation mode for each subject were performed. If the leg did not perform as expected, the user re-attempted the activity without any changes made to the DNN model. After several attempts, if the therapist judged that completion of the activity was not possible for the user, that activity was not attempted anymore.

### 2.5. Data analysis

The performance of the proposed DNN model was evaluated in two aspects: offline testing data analysis and online testing data analysis. For offline testing analysis, we computed the root mean square difference (RMSD) between impedance parameters: those from the state machine that controlled the leg and those estimated using the proposed DNN. To evaluate the differences in the impedance parameters between the state machine and DNN, the coefficient of determination ( 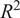 ) was computed as follows:(3)

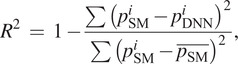

where 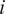 represents the impedance, 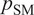 and 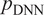 denote trajectory of individual impedance parameters (i.e., 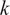 , 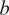 , or 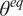 ) obtained from the state machine and the DNN model, respectively. 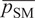 denotes the mean of the state machine-based impedance parameter.

For online testing analysis, the ratio between the number of successful steps and total steps was computed. A successful step was categorized as a step where a natural gait pattern, judged by the therapist who was supervising the subject, occurred.

In addition, latent and time sequence features of the proposed DNN for online testing were investigated to verify that the DNN can properly extract information regarding ambulation modes and gait phase from sensor data. To visualize the distribution of latent features, t-distributed stochastic neighbor embedding (t-SNE, Van der Maaten and Hinton, 2008) was employed for dimension reduction. To evaluate the correlation of time sequence features across ambulation modes, Pearson correlation coefficients between time sequence feature sets (i.e., 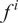 and 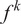 ) were obtained as follows:(4)

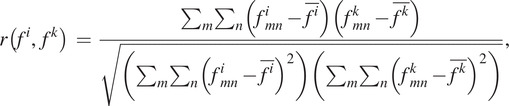

where 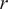 denotes correlation coefficient, 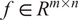 denotes the time sequence feature set, 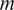 denotes the number of time sequence features (i.e., 12 in this study, see Figure 1d), 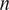 denotes the length of the feature (i.e., [0–100]% gait cycle), and 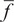 denotes mean of the time sequence feature set.

To evaluate the statistical differences, two-sample *t*-tests were performed using the assumption that differences are from normal distributions with unknown and unequal variances.

## 3. Results

### 3.1. Offline testing

**Figure 3.**
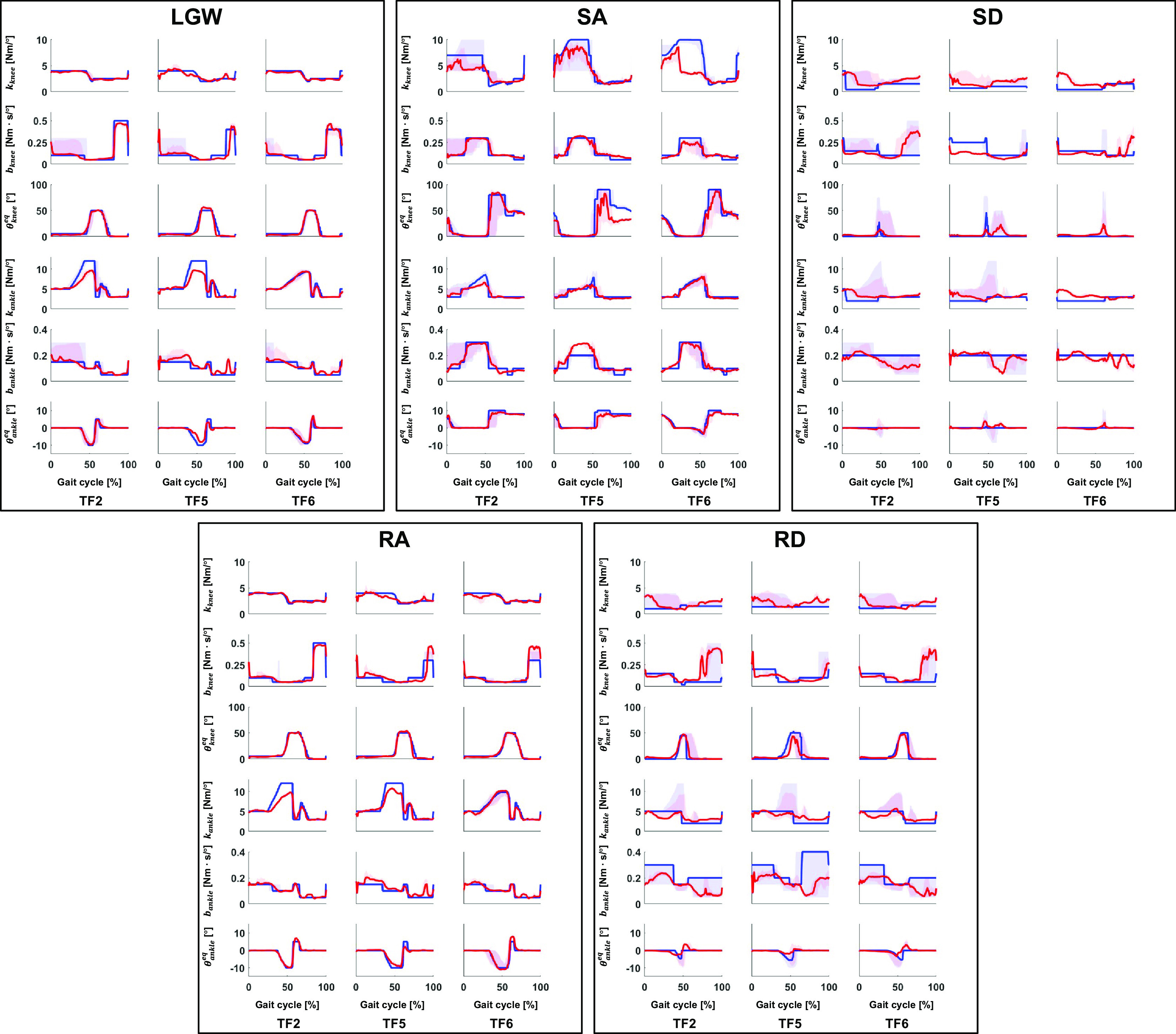
Comparison of impedance parameters generated from the state machine (blue lines) and DNN (red lines) in offline tests. In general, the DNN made impedance parameters similar to the state machine for the user who was used to train the DNN model (i.e., TF2) and the new users (i.e., TF5 and TF6). The median 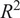 across all impedance parameters for all ambulation modes were 0.77, 0.46, and 0.79 for TF2, TF5, and TF6, respectively; the median 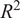 for all users was 0.72. All plots show 75th and 25th percentiles in lighter bands.

**Figure 4.**
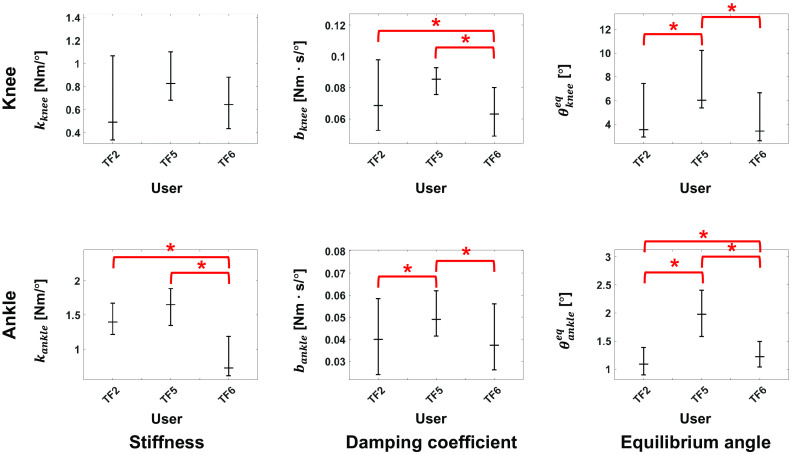
RMSD of all five ambulation modes in offline tests. The users who were not used for the training (i.e., TF5 and TF6) showed a similar error level as the user who was used for training (i.e., TF2). Bar plots show the 25th, 50th (median), and 75th percentiles. Asterisk indicates 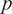 < .05.

Figures 3 and 4 show the fitted offline impedance parameters and their RMSD, respectively. The median 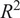 across all the impedance parameters for all ambulation modes between the DNN model and the state machine for the user who was used to train the model (i.e., TF2) and the users who were not used to train the DNN model (i.e., TF5 and TF6) were 0.77, 0.46, and 0.79, respectively.

Although TF5 had the largest deviation, there was no difference in the RMSD between TF2, TF5, and TF6 at a 5% significance level for impedance parameters except the ankle equilibrium angle, as shown in Figure 4. Thus, the proposed DNN showed consistent performance characteristics for the new and trained users.

### 3.2. Online testing

**Figure 5.**
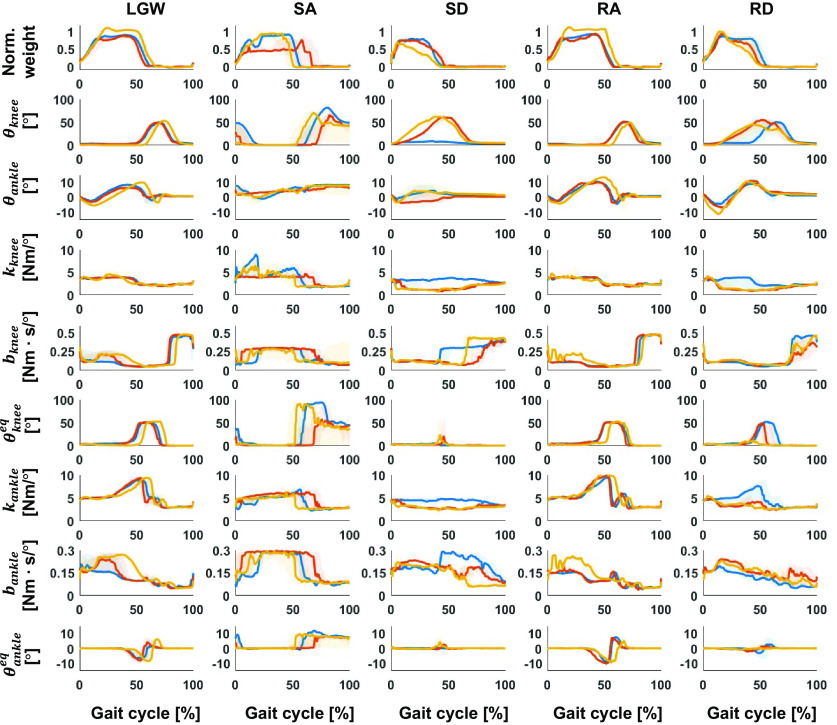
Gait trajectory and corresponding impedance parameters across five ambulation modes during online testing. The blue, red, and yellow lines represent TF2, TF3, and TF5, respectively. All plots show 75th and 25th percentiles in lighter bands.

During ambulation, the DNN generated the impedance parameters for all configured activities (Figure 5). These parameters allowed the subjects to ambulate successfully in most trials (Table 5), with an overall success rate of 96%. However, we found that some users had problems performing specific activities. TF2 had difficulty in performing SD. She could not bend the knee, and the leg remained too stiff to descend the stairs using a reciprocal gait pattern. TF3 had difficulty in performing SA. The leg remained in an LGW even though the user wished to transition into a SA activity. Qualitatively, the users felt differences between the state machine and proposed DNN approach, but they did not consistently choose one method over the other. Some notes from users follow: TF2 preferred the DNN because she said it generated more natural motions in LGW and RD; TF3 stated he felt less support (i.e., a looser knee joint) in RD when using DNN; and TF5 stated he felt more support (i.e., a stiffer knee joint) in SD and RD.

**Table 5.** Successful motions during online testing with respect to total trials

	Successful motions/total trials (success rate)					
User	LGW	SA	SD	RA	RD	Total
TF2	93/93 (100%)	21/23 (91%)	0/6 (0%)	89/90 (99%)	98/101 (97%)	301/313 (96%)
TF3	101/102 (99%)	9/15 (60%)	21/21 (100%)	25/25 (100%)	27/27 (100%)	183/190 (96%)
TF5	166/173 (96%)	13/18 (72%)	16/16 (100%)	13/13 (100%)	21/21 (100%)	229/241 (95%)
Total	360/368 (98%)	43/56 (77%)	37/43 (86%)	127/128 (99%)	146/149 (98%)	713/744 (96%)

Abbreviations: LGW, level-ground walking; RA/RD, ascending/descending a ramp; SA/SD, ascending/descending stairs.

### 3.3. Feature extraction

**Figure 6.**
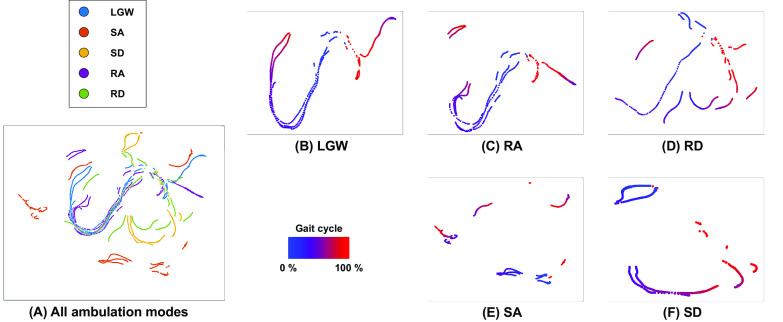
Visualization of latent features using t-SNE from the online test dataset. (a) Feature distributions for all ambulation modes. Features are clearly separated except for LGW and RA because their characteristics are similar. (b)–(f) present feature distributions of individual ambulation modes. The feature distributions have long and curved shapes because the features represent a continuous gait trajectory.

The proposed DNN architecture extracts latent and time sequence features; the combination of latent and time sequence features allows the system to distinguish user intention across five ambulation modes and to generate the desired impedance parameters. The latent features shown in this section are a result of performing t-SNE from data recorded during the online testing. LGW and RA have similar gait trajectories (Figure 6). By contrast, SA has the most distinctive trajectory among the modes. The visualized features have long and curved shapes, as shown in Figure 6b–f. Although the axes in t-SNE have no physical meaning, we can presume that the extracted features represent a continuous gait trajectory.

**Figure 7.**
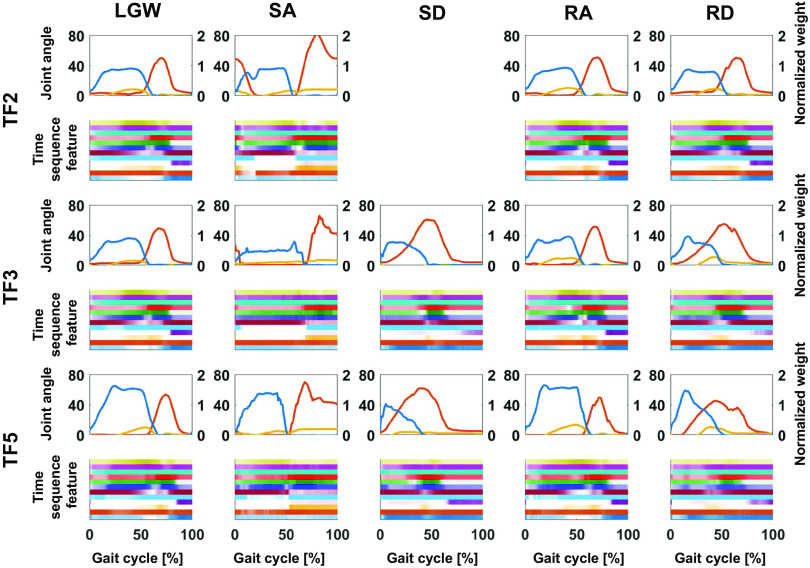
Median gait trajectory (upper figures) and corresponding time sequence feature distribution (lower figures) during online testing. The blue, yellow, and red lines in the upper figures represent the normalized weight, ankle angle, and knee angle, respectively. Each color in the lower figures represents the activation level of each time sequence feature. The time sequence features were normalized with respect to the maximum and minimum. The features were sensitively changed according to the gait phase.

**Figure 8.**
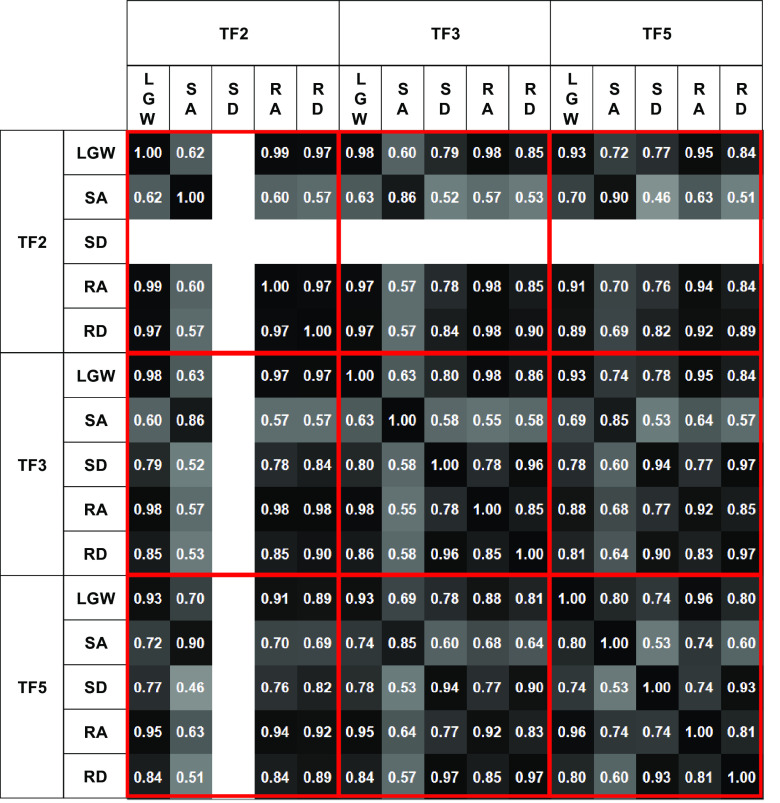
Feature correlation with respect to ambulation modes and users. High correlation (minimum of 0.46 and median of 0.82) indicates that the time sequence features are sensitive to the gait phase, rather than ambulation mode. SD for TF2 was excluded because TF2 was unable to complete the mode.

Figure 7 presents the time sequence features extracted from the online test. The time sequence features were sensitive and changed according to the gait phases regardless of the ambulation modes. For example, the fourth feature from the bottom (purple line) was activated at the end of the gait cycle for all ambulation modes, except for SA; the fourth feature from the top (red line) was more activated in the swing phase than in the stance phase. Therefore, time sequence features may contain phase information for each ambulation mode. Furthermore, the time sequence features had a high correlation (median of 0.82) for all users across all ambulation modes, as shown in Figure 8. These results indicate that the time sequence features are sensitive to the gait phases and are less sensitive to ambulation modes. In other words, the time sequence features contain information about the gait phase.

In conclusion, the proposed DNN model generated the impedance parameters by identifying the ambulation mode and gait phase through the latent and main networks, respectively.

### 3.4. Gait comparison

**Figure 9.**
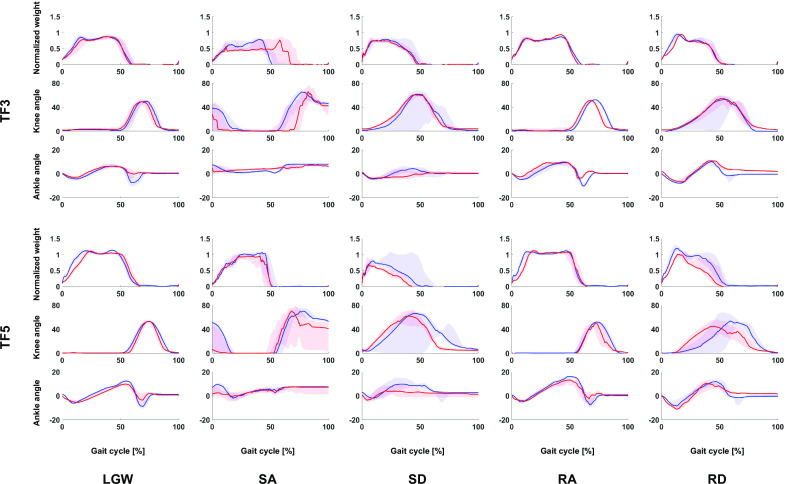
Gait comparison of offline (blue lines) and online data (red lines). All plots show 75th and 25th percentiles in lighter bands.

**Figure 10.**
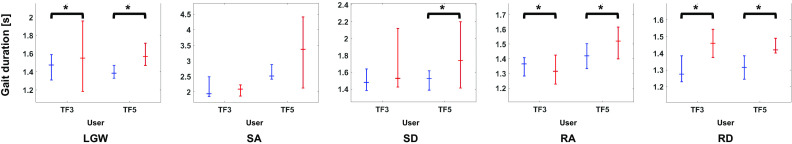
Comparison of gait duration between offline data (blue lines) and online data (red lines) for five ambulation modes. In general, the DNN results in longer gait duration than the state machine. Bar plots show the 25th, 50th (median), and 75th percentiles. Asterisk indicates 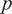 < .05.

TF3 and TF5 participated in both the offline and online tests. Their gait trajectories are presented in Figure 9. In general, they show similar gait trajectory shapes. While the shape was similar, the timing of the DNN was typically longer and more variable (Figure 10). We speculate that the DNN model trained on a variety of users has more gait variability than the user-specific state machine.

## 4. Discussion

The DNN model extracts time sequence features and latent features representing information regarding the gait phase and intended ambulation modes to learn the inherent structure of the state machine, including impedance parameter values, within ambulation mode state-transitions and between ambulation mode state-transitions. Though the model was only trained from a relatively small number of subjects who walked a combined total of 5,417 steps, the method showed promising results, even when tested by a novel subject.

The DNN training data used in the study was based on data generated when subjects were ambulating with state machine control. The input data were sensor readings, and the output data were impedance parameters. This approach could be applied to different input/output mappings. For example, it would be possible to perhaps map the input sensors to motor currents, bypassing the need to use finite-state machine-based impedance control. In such a situation, it could be possible to incorporate different mid-level control approaches into the overall control scheme. For example, the gait-phase-based control style proposed by Gregg’s group (Quintero et al., 2016) could be used to generate training data for walking, while an impedance-based controller could be used to generate training data for walking on stairs. When applying the DNN approach, the resulting controller could learn attributes from both the phase-based and impedance-based control approaches. This remains to be tested.

The impedance parameters included in the state machine should not be considered as ground truth or optimal. They were configured to allow safe and comfortable walking. The parameters predicted by the DNN are different. Still, they should, in most cases, not be considered better or worse, with the exception of the situations where the users could not perform the activity. This is reinforced by the qualitative feedback from the users who reported that they felt differences between the two methods, but most noted that it was difficult to say which method, if either, was preferred. However, as shown in Figure 10, there was a difference in terms of gait duration between the two methods. Detailed comparisons that consider variable factors, such as metabolic costs, may be needed to verify which is better.

In the future, a more detailed and quantitative analysis should be conducted to investigate the performance of the DNN in terms of the naturalness and intuitiveness of the gait. We expect that the performance can be improved with more training datasets since the current model was trained with a dataset combined from only four transfemoral amputee users. Including data from more users as well as users performing a wider variety of postures to initiate movement, including a greater variability within each ambulation mode, may allow the model to recognize a higher percentage of tasks. Our study had several limitations. The collected data were unbalanced, and no data sampling method was used to compensate for the imbalance. There are far more data available for LGW than the sum of the rest ambulation modes. Although this may be likely consistent with real-world use (i.e., ambulation in LGW occurs with much higher frequency than on stairs and ramps), there may be a risk that the model parameters will not properly generate motion for stairs and ramps. In other words, the model parameters can be trained by focusing only on LGW.

TF2 and TF3 had more steps than other users for training. A large amount of data obtained from a particular user can lead to model bias since the model parameters may be overfitted to specific physical characteristics. For instance, the users have different stride lengths. Ascending ramps of the same distance (i.e., a 14-foot ramp) took about 9, 6, and 4 steps for TF2, TF3, and TF5, respectively. This imbalance may have produced a biased model working for users who have similar physical characteristics to users for training. The low 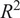 obtained for TF5 in offline testing (Section 3.1) may be induced by the model bias since the height and weight of TF5 were significantly different from those of other users, as shown in Table 3.

Prior to testing unilateral transfemoral users, we performed bypass testing with four non-disabled individuals for model validation. This study used 50 ms of sensor data history to generate the impedance parameters. Although we investigated models that use a longer history (e.g., 75, 100, or 150 ms), there was no difference in performance based on visual inspections of gait. Thus, we chose the 50 ms history to reduce computation cost. The latent network was crucial to generating impedance parameters for the ambulation modes; the DNN model tended to generate parameters only for LGW when the latent network was not used. We speculate that the unbalanced data caused this problem. Time sequence features in the main network were extracted from three of 18 sensors (i.e., weight, thigh angle, and shank angle) because we believe this data directly represents lower limb movement. In addition, when we included knee and ankle angles in the input of the main network, the model generated equilibrium angles very close to the current knee and ankle angles; the leg did not move during the online testing. We speculate that the model predicted the equilibrium angles using only the current joint angles (i.e., knee and ankle). Therefore, the model may have tried to maintain the current stationary joint angle instead of generating a trajectory to restore locomotion.

Ideally, we would have included more data in our analysis and testing. The COVID-19 pandemic resulted in many subjects canceling visits. In spite of this, we were still able to collect a sufficient amount of data to demonstrate the feasibility of the approach with online evaluation. Future research will require experiments under well-organized conditions: considering a wider variety of subjects (i.e., stride length, height, weight, and natural speed) and under various environments (i.e., stairs with different heights and ramps with different slopes).

## 5. Conclusion

In this study, we designed and tested DNN-based powered prosthetic leg control for unilateral transfemoral amputee users. The proposed DNN model was trained with four subjects and applied in offline and online tests with four subjects. The DNN successfully generated impedance parameters for the gait across various ambulation modes and enabled seamless transitions.

The proposed DNN consists of two sub-networks: the latent and main networks. Their combination can distinguish user intention for all five ambulation modes and gait phases. However, further investigation is needed because there were wrong step motions during SA; more specifically, the DNN generated LGW motions in some cases instead. In addition, although participant TF2 was used to train the DNN, she could not perform SD. We believe that testing with big data will result in more reliable gait generation.

In conclusion, the proposed DNN can facilitate its application to any patient because it does not require tuning; parameter fitting for state machines is time-consuming and inconvenient for prosthetists/therapists and patients. This training-free and intuitive control method that works across different ambulation modes will help develop technologies to improve the quality of life for lower limb amputees.

## Data Availability

The datasets used and/or analyzed during the current study are available from the corresponding author upon reasonable request.

## Supplementary material

For supplementary material accompanying this paper visit https://doi.org/10.1017/wtc.2022.19. click here to view supplementary material
