# Supplementary material for: Seamless and intuitive control of a powered prosthetic leg using deep neural network for transfemoral amputees
Source: Wearable Technol. 2022 Sep 28;3:e24. doi: 10.1017/wtc.2022.19 (PMC10085575; doi:10.1017/wtc.2022.19)
Supplement: Supplementary file 1 [file S2631717622000196sup001.pdf]

Subject: **TF1**

Mode	Phase	Knee parameters			Ankle parameters		
		k [Nm/deg]	b [Nm·s/deg]	θ^{eq} [deg]	k [Nm/deg]	b [Nm·s/deg]	θ^{eq} [deg]
**Walk**	Early Stance	4	[0.1, 0.4]	5	[5, 12]	0.15	[-10, 0]
	Late Stance	[2, Prev.]	0.05	[Prev., 50]	[Prev., 12]	0.1	[-6, Prev.]
	Early Swing	2.5	0.05	50	3	0.15	5
	Late Swing	[2.5, Prev.]	[0.1, 0.5]	[0, Prev.]	[3, 8]	0.05	0
**Ramp Ascent**	Early Stance	1.1	0.15	0	5	0.3	0
	Late Stance	1	0.05	[Prev., 55]	5	0.15	[-5, Prev.]
	Early Swing	1.7	0.02	55	2	0.15	0
	Late Swing	1.5	0.05	0	2	0.2	0
**Ramp descent**	Early Stance	1.1	0.15	0	5	0.3	0
	Late Stance	1	0.05	[Prev., 55]	5	0.15	[-5, Prev.]
	Early Swing	1.7	0.02	55	2	0.15	0
	Late Swing	1.5	0.05	0	2	0.2	0
**Stair Ascent**	Stance	[7, 10]	0.1	[0, Prev.]	3	0.1	[0, Prev.]
	Early Swing	[1, 2]	0.15	110	3	0.1	10
	Midswing	2	0.05	50	3	0.05	8
	Late Swing	2.5	0.05	[0, 90]	3	0.1	8
**Stair descent**	Early Stance	0.4	0.1	0	2	0.2	0
	Late Stance	0.4	0.1	0	2	0.2	0
	Early Swing	0.7	0.25	Pknee+10	1.5	0.2	Pankle
	Late Swing	1.5	0.1	0	3	0.2	0

Prev. denotes the final value in the previous state.
Pknee denotes the final knee angle in the previous state.
Pankle denotes the final ankle angle in the previous state.

Subject: **TF2**

Mode	Phase	Knee parameters			Ankle parameters		
		k [Nm/deg]	b [Nm·s/deg]	θ^{eq} [deg]	k [Nm/deg]	b [Nm·s/deg]	θ^{eq} [deg]
**Walk**	Early Stance	4	[0.1, 0.4]	5	[5, 9.8]	0.15	[-12, 0]
	Late Stance	[2, Prev.]	0.05	[Prev., 40]	[Prev., 9.8]	0.1	[-6, Prev.]
	Early Swing	2	0.05	40	3	0.15	5
	Late Swing	[1.4, Prev.]	[0.05, 0.4]	[0, Prev.]	[5, 8]	0.05	0
**Ramp Ascent**	Early Stance	4	[0.1, 0.4]	5	[5, 9.8]	0.15	[-12, 0]
	Late Stance	[2, Prev.]	0.05	[Prev., 40]	[Prev., 9.8]	0.1	[-12, 0]
	Early Swing	2	0.05	40	3	0.15	5
	Late Swing	[1.6, Prev.]	[0.05, 0.3]	[0, Prev.]	[5, 8]	0.05	0
**Ramp descent**	Early Stance	1.1	0.2	0	5	0.4	0
	Late Stance	1.1	0.05	[Prev., 55]	5	0.15	[-6, Prev.]
	Early Swing	1.2	0.05	55	2	0.15	0
	Late Swing	1.2	0.1	0	2	0.2	0
**Stair Ascent**	Stance	[7, 8]	0.15	[0, Prev.]	2.5	0.05	[0, Prev.]
	Early Swing	1	0.05	100	3	0.2	15
	Midswing	1.5	0.05	65	3	0.1	5
	Late Swing	1.5	0.05	[0, 90]	3	0.1	5
**Stair descent**	Early Stance	0.4	0.25	0	2	0.2	0
	Late Stance	0.4	0.25	0	2	0.2	0
	Early Swing	0.5	0.3	Pknee+10	1.5	0.2	Pankle
	Late Swing	1	0.1	0	3	0.2	0

Prev. denotes the final value in the previous state.
Pknee denotes the final knee angle in the previous state.
Pankle denotes the final ankle angle in the previous state.

Subject: **TF3**

Mode	Phase	Knee parameters			Ankle parameters		
		k [Nm/deg]	b [Nm·s/deg]	θ^{eq} [deg]	k [Nm/deg]	b [Nm·s/deg]	θ^{eq} [deg]
**Walk**	Early Stance	4	[0.1, 0.4]	5	[5, 9.8]	[0.1, 0.2]	[-13, 0]
	Late Stance	[2, Prev.]	0.05	[Prev., 40]	[Prev., 9.8]	0.1	[-6, Prev.]
	Early Swing	2	0.05	40	3	0.15	5
	Late Swing	[1.6, Prev.]	[0.05, 0.3]	[0, Prev.]	[3, 8]	0.05	0
**Ramp Ascent**	Early Stance	4	[0.1, 0.4]	5	[3, 9.8]	[0.1, 0.2]	[-15, 0]
	Late Stance	[2, Prev.]	0.05	[Prev., 40]	[Prev., 9.8]	0.1	[-15, 0]
	Early Swing	2	0.05	40	3	0.15	5
	Late Swing	[1.3, Prev.]	[0.05, 0.3]	[0, Prev.]	[3, 8]	0.05	0
**Ramp descent**	Early Stance	1.2	0.2	0	5	0.4	0
	Late Stance	1.2	0.05	[Prev., 55]	5	0.15	[-6, Prev.]
	Early Swing	1.2	0.05	55	2	0.15	0
	Late Swing	1.2	0.1	0	2	0.2	0
**Stair Ascent**	Stance	[7, 9]	0.05	[0, Prev.]	2.5	0.05	[0, Prev.]
	Early Swing	1	0.05	90	3	0.2	15
	Midswing	1.5	0.05	65	3	0.1	8
	Late Swing	1.5	0.05	[0, 90]	3	0.1	8
**Stair descent**	Early Stance	0.6	0.15	0	2	0.2	0
	Late Stance	0.6	0.15	0	2	0.2	0
	Early Swing	0.5	0.3	Pknee+10	1.5	0.2	Pankle
	Late Swing	1	0.1	0	3	0.2	0

Prev. denotes the final value in the previous state.
Pknee denotes the final knee angle in the previous state.
Pankle denotes the final ankle angle in the previous state.

Subject: **TF4**

Mode	Phase	Knee parameters			Ankle parameters		
		k [Nm/deg]	b [Nm·s/deg]	θ^{eq} [deg]	k [Nm/deg]	b [Nm·s/deg]	θ^{eq} [deg]
**Walk**	Early Stance	4	[0.1, 0.4]	5	[5, 9.8]	0.15	[-12, 0]
	Late Stance	[2, Prev.]	0.05	[Prev., 40]	[Prev., 9.8]	0.1	[-6, Prev.]
	Early Swing	2	0.05	40	3	0.15	5
	Late Swing	[1.3, Prev.]	[0.05, 0.3]	[0, Prev.]	[5, 8]	0.15	0
**Ramp Ascent**	Early Stance	4	[0.1, 0.4]	5	[5, 9.8]	0.15	[-12, 0]
	Late Stance	[2, Prev.]	0.05	[Prev., 40]	[Prev., 9.8]	0.1	[-12, 0]
	Early Swing	2	0.05	40	3	0.15	5
	Late Swing	[1.4, Prev.]	[0.05, 0.3]	[0, Prev.]	[5, 8]	0.05	0
**Ramp descent**	Early Stance	1.2	0.2	0	5	0.4	0
	Late Stance	1.2	0.05	[Prev., 55]	5	0.15	[-6, Prev.]
	Early Swing	1.2	0.05	55	2	0.15	0
	Late Swing	1.2	0.1	0	2	0.2	0
**Stair Ascent**	Stance	[7, 8]	0.15	[0, Prev.]	2.5	0.05	[0, Prev.]
	Early Swing	1	0.05	100	3	0.2	15
	Midswing	1.5	0.05	65	3	0.1	8
	Late Swing	1.5	0.05	[0, 90]	3	0.1	8
**Stair descent**	Early Stance	0.6	0.25	0	2	0.2	0
	Late Stance	0.6	0.25	0	2	0.2	0
	Early Swing	0.5	0.3	Pknee+10	1.5	0.2	Pankle
	Late Swing	1	0.1	0	3	0.2	0

Prev. denotes the final value in the previous state.
Pknee denotes the final knee angle in the previous state.
Pankle denotes the final ankle angle in the previous state.

Subject: **TF5**

Mode	Phase	Knee parameters			Ankle parameters		
		k [Nm/deg]	b [Nm·s/deg]	θ^{eq} [deg]	k [Nm/deg]	b [Nm·s/deg]	θ^{eq} [deg]
**Walk**	Early Stance	4	[0.1, 0.4]	5	[Prev., 9.5]	[0.1, 0.2]	0
	Late Stance	[2, Prev.]	0.05	[Prev., 50]	[Prev., 9.5]	0.1	[-6, Prev.]
	Early Swing	2	0.05	50	3	0.15	5
	Late Swing	[1.3, Prev.]	[0.1, 0.3]	[0, Prev.]	[3, 8]	0.05	0
**Ramp Ascent**	Early Stance	4	[0.1, 0.4]	5	[Prev., 9.8]	[0.1, 0.2]	0
	Late Stance	[2, Prev.]	0.05	[Prev., 50]	[Prev., 9.5]	0.1	[-6, Prev.]
	Early Swing	2	0.05	50	3	0.15	5
	Late Swing	[1.3, Prev.]	[0.1, 0.3]	[0, Prev.]	[3, 8]	0.05	0
**Ramp descent**	Early Stance	1.1	0.2	0	5	0.4	0
	Late Stance	1.1	0.05	[Prev., 55]	5	0.15	[-6, Prev.]
	Early Swing	1.2	0.05	55	2	0.15	0
	Late Swing	1	0.1	0	2	0.2	0
**Stair Ascent**	Stance	9	0.05	[0, Prev.]	2.5	0.05	[0, Prev.]
	Early Swing	1	0.05	100	3	0.2	15
	Midswing	1.5	0.05	65	3	0.1	8
	Late Swing	1.5	0.05	[0, 90]	3	0.1	8
**Stair descent**	Early Stance	0.7	0.25	0	2	0.2	0
	Late Stance	0.7	0.25	0	2	0.2	0
	Early Swing	0.5	0.3	Pknee+10	1.5	0.2	Pankle
	Late Swing	1	0.1	0	3	0.2	0

Prev. denotes the final value in the previous state.
Pknee denotes the final knee angle in the previous state.
Pankle denotes the final ankle angle in the previous state.

Subject: **TF6**

Mode	Phase	Knee parameters			Ankle parameters		
		k [Nm/deg]	b [Nm·s/deg]	θ^{eq} [deg]	k [Nm/deg]	b [Nm·s/deg]	θ^{eq} [deg]
**Walk**	Early Stance	4	[0.1, 0.4]	5	[5, 12]	0.15	[-10, 0]
	Late Stance	[2, Prev.]	0.05	[Prev., 50]	[Prev., 12]	0.1	[-10, Prev.]
	Early Swing	2.5	0.05	50	3	0.15	5
	Late Swing	[2.5, Prev.]	[0.1, 0.4]	[0, Prev.]	[3, 8]	0.05	0
**Ramp Ascent**	Early Stance	4	[0.1, 0.4]	5	[5, 12]	0.15	[-10, 0]
	Late Stance	[2, Prev.]	0.05	[Prev., 50]	[Prev., 12]	0.1	[-10, Prev.]
	Early Swing	2.5	0.05	50	3	0.15	5
	Late Swing	[2.5, Prev.]	[0.05, 0.3]	[0, Prev.]	[3, 8]	0.05	0
**Ramp descent**	Early Stance	1.1	0.15	0	5	0.3	0
	Late Stance	1.2	0.05	[Prev., 55]	5	0.15	[-6, Prev.]
	Early Swing	1.7	0.02	55	2	0.15	0
	Late Swing	1.5	0.05	0	2	0.2	0
**Stair Ascent**	Stance	[9, 12]	0.1	[0, Prev.]	3	0.1	[0, Prev.]
	Early Swing	[1, 2]	0.1	90	3	0.1	10
	Midswing	[1, 2]	0.05	40	3	0.1	8
	Late Swing	2.5	0.05	[0, 90]	3	0.1	8
**Stair descent**	Early Stance	0.4	[0.15, 0.3]	0	2	0.2	0
	Late Stance	0.4	[0.15, 0.3]	0	2	0.2	0
	Early Swing	0.7	0.4	Pknee+10	1.5	0.2	Pankle
	Late Swing	1.5	0.1	0	3	0.2	0

Prev. denotes the final value in the previous state.
Pknee denotes the final knee angle in the previous state.
Pankle denotes the final ankle angle in the previous state.
